# CNS‐active drugs for neuropsychiatric disorders differentially modulate the risk of AD development

**DOI:** 10.1002/alz.091759

**Published:** 2025-01-03

**Authors:** Helena Cortes‐Flores, Georgina Torrandell‐Haro, Roberta Diaz Brinton

**Affiliations:** ^1^ University of Arizona, Tucson, AZ USA; ^2^ PO Box 210242, Tucson, AZ USA

## Abstract

**Background:**

Neuropsychiatric disorders including depression, insomnia, epilepsy, schizophrenia, and attention‐deficit and hyperactivity disorder (ADHD) have been associated with a neurodegenerative process and linked to increased risk for Alzheimer’s Disease (AD). Because of the shared biological mechanisms of AD and neuropsychiatric disorders, we hypothesized that pharmacologic treatment for neuropsychiatric disorders could impact the risk for AD. CNS drugs that are first‐line therapies for neuropsychiatric disorders (including antidepressants, sedatives, anticonvulsants, antipsychotics, and stimulants) were investigated for impact on AD incidence.

**Method:**

To address this hypothesis, we conducted a retrospective medical informatics analysis of insurance claims of patients aged 60 years and older, with and without exposure to CNS drugs. To reduce health status and demographic bias, we utilized propensity score matching to adjust for age, gender, Charlson comorbidity index (CCI), and comorbidities. The propensity score matched population was surveyed for AD diagnosis following at least 1 year of exposure to CNS drugs.

**Result:**

Exposure to CNS drugs was associated with a decreased risk for AD (RR [95%CI]: 0.50 [0.47–0.53]; *P*<.0001), and women (RR [95%CI]: 0.46 [0.42–0.50]; *P*<.0001) exhibited a slightly greater risk reduction compared to men (RR [95%CI]: 0.55 [0.50–0.61]; *P*<.0001). Drug stratification indicated that antidepressant, sedative, anticonvulsant, and stimulant treatment were associated with reduced AD risk, while antipsychotics were associated with increased risk for AD. Additional analysis indicated that the combination of two CNS drugs was associated with a greater reduction of AD risk compared to monotherapy for most drugs assessed. Age and sex emerged as modulators of rate of disease conversion, with females older than 70 years receiving the greatest benefit.

**Conclusion:**

Collectively, these results indicate that antidepressants, sedatives, anticonvulsants, and stimulants are beneficial as risk modifiers for AD, while antipsychotics appear to increase the vulnerability of the brain to AD development. The potential use of combination therapy with antipsychotics could mitigate the risk conferred by these drugs. These findings have the potential to contribute to advancing neuropsychiatric treatment that could impact the risk of Alzheimer’s disease.

